# Sugar accelerates chronological aging in yeast via ceramides

**DOI:** 10.15698/cst2025.07.308

**Published:** 2025-07-22

**Authors:** Vera Schmiedhofer, Julian Sommersguter-Wagner, Oskar Knittelfelder, Helmut Jungwirth, Gerald N. Rechberger, Didac Carmona-Gutierrez, Patrick Rockenfeller, Christoph Ruckenstuhl, Frank Madeo

**Affiliations:** 1 Institute of Molecular Biosciences, University of Graz, NAWI Graz, Graz, Austria.; 2 Max Planck Institute of Molecular Cell Biology and Genetics, Dresden, Germany.; 3 Center for society, knowledge and communication (7th faculty), University of Graz, Graz, Austria.; 4 Field of Excellence BioHealth-University of Graz, Graz, Austria.; 5 Chair of Biochemistry and Molecular Medicine, Center for Biomedical Education and Research (ZBAF), University of Witten/Herdecke (UW/H), Witten, Germany.; 6 BioTechMed Graz, Graz, Austria.; a These authors contributed equally.

**Keywords:** aging, metabolic syndrom, cell stress, nutrient signaling, lipotoxicity, chronological lifespan, VAC8, TOR1, lipid homeostasis

## Abstract

High carbohydrate intake, a characteristic of many Western diets, is a major contributor to age-associated pathologies. Here, we explored the molecular consequences of sugar overload during chronological aging in the yeast *Saccharomyces cerevisiae*. High levels of glucose and fructose resulted in a decrease of chronological lifespan as well as an increase of cell death, ROS and neutral lipids. Interestingly, these changes were accompanied by significantly altered ceramide profiles. Deletion of either the kinase Tor1, a master regulator of growth and autophagy in response to nutrients, or the vacuole-anchored receptor Vac8, an important player in various autophagy pathways, improved survival and normalized ceramide profiles. This suggests that ceramides might play a role in sugar stress-induced cell death. In line, pharmacological inhibition of sphingolipid synthesis normalized ceramide profiles and improved chronological lifespan, whereas pharmacologically induced ceramide accumulation decreased chronological lifespan. In sum, our findings causally link nutrient signaling and an altered ceramide profile to sugar cytotoxicity in aging yeast, providing a basis for further search of feasible interventions against sugar-induced cell death.

## Abbreviations

AbA - Aureobasidin A

AV - Annexin V

CLS - chronological lifespan

CVD - cardiovascular disease

Cvt - cytoplasm to vacuole targeting

LCBs - long-chain bases

NVJ - vacuole nucleus junctions

PC - phosphatidylcholine

PE - phosphatidylenthanolamine

PI - propidium iodide

PMN - piecemeal microautophagy of the nucleus

ROS - reactive oxygen species

SPT - serine palmitoyltransferase

TOR - target of rapamycin

TORC1 - TOR-complex 1.

## INTRODUCTION

Today, obesity is not an exclusive problem to affluent western societies, but has risen to a global pandemic with more people being obese than underweight. The worldwide prevalence of obesity has almost tripled within the last 40 years, a dramatic development that affects both adults and children 1. This has resulted not only in a great socioeconomic burden, but also poses a huge challenge to the health care systems. One of the main reasons for that is that obesity is a risk factor for many - often age-related - pathologies, such as cardiovascular diseases (CVDs), diabetes, musculoskeletal disorders and some cancers. A meta-analysis revealed that 5-unit increase in BMI above 25 kg/m^2^ increases overall mortality by 29%, cardiovascular mortality by 41% and mortality related to diabetes by 210% 2. Nutritional changes around the globe play an important role in such obesity pandemic. In this regard, sugar consumption has tripled worldwide over the past 50 years, among others multiplying cardiovascular mortality 2-3 times in people consuming 10% or more of total calories (based on 2,000 kcal/day) from added sugars 3. High sugar consumption has not only been linked to obesity, but also to an increased incidence of metabolic disease, even in people with normal weight 4. Recent data have substantiated the notion that sugar overconsumption accelerates the progression of the aging process 5,6,7,8,9,10,11. In line, epidemiological studies clearly indicate that high sugar intake is a driving force in the development of life-threatening conditions such as type II diabetes, CVDs and cancer 12. Still, the detrimental consequences of sugar-overload at the cellular level, ultimately resulting in cellular demise, remain poorly studied. In order to address this gap, we decided to investigate sugar toxicity during aging by making use of the yeast *Saccharomyces cerevisiae* as a model organism. Yeast chronological aging (hereafter referred to as "aging") is an established paradigm 13, where the chronological lifespan (CLS) is defined as the time cells survive in the post-diauxic and stationary phase 14. Thus, it most commonly serves as model for the aging of post-mitotic human tissue cells 15. Besides its prolific history as an aging model, *S. cerevisae* has served as a versatile model organism to study regulated cell death mechanisms, including apoptosis 16,17,18,19,20,21,22.

At the same time, work in *S. cerevisiae* has made fundamental contributions to our understanding of sugar-related energy-metabolism 23, most notably, the elucidation the glycolysis process itself, which was mostly deciphered in yeasts during the last half of the 19^th^ and the first half of the 20^th^ century 24. However, it was not until the late 1990s, when several research groups started examining sugar-related (or glucotoxic) stress in *S. cerevisiae* that allowed establishing a connection to different aspects of aging and cell death mechanisms. For instance, it has been demonstrated that glucose induces cell death of stationary-phase yeast cells only within a few hours, if additional nutrients are absent, whereas cells incubated in water (without sugar addition) stay viable for weeks 25,26. Sugar-induced lethal stress was shown to exhibit apoptotic markers like DNA degradation, cell shrinkage and nuclear fragmentation. In addition, cell death seems to be accompanied by an accumulation of reactive oxygen species (ROS) and to depend on hexose phosphorylation 27,28. Beyond yeast, high-glucose intake has been shown to decrease lifespan in both the nematode *Caenorhabditis elegans* and mice 29,30,31,32. Several studies have also revealed that a sucrose-rich diet leads to the development of insulin resistance and additional obesity in mice and rats 33,34,35. Moreover, in nonhuman primates, high fructose intake rapidly leads to hepatic steatosis, or fatty liver disease, without weight gain. Thereby, fructose overconsumption seems to compromise intestinal protection, causing a detrimental increase (30%) of bacterial migration to the liver 36.

Despite these fundamental observations into the adverse effects of high sugar diets, detailed mechanistic insights into the underlying cellular pathways remain rare.

In this work, we investigated the molecular determinants implicated in the cytotoxic effects upon glucose and fructose overload during chronological aging in yeast. Glucose and fructose represent the two mono-saccharides that build-up sucrose, the most commonly used sugar world-wide. We found that high levels of each of these sugars reduce CLS, which was accompanied by significantly increased levels of diacylglycerol (DAG), triacylglycerol (TAG) and ceramides. In addition, we identified the kinase Tor1 and the autophagy- and vacuole-related protein Vac8 as well as the process of sphingolipid synthesis to be causal mediators of sugar-induced cell death during aging. Altogether this study presents novel evidence that links sugar cytotoxicity to specific molecular instances related to nutrient control and lipid metabolism.

## RESULTS

### High concentrations of glucose and fructose exhibit cytotoxic effects upon chronological aging

**Figure 1:**
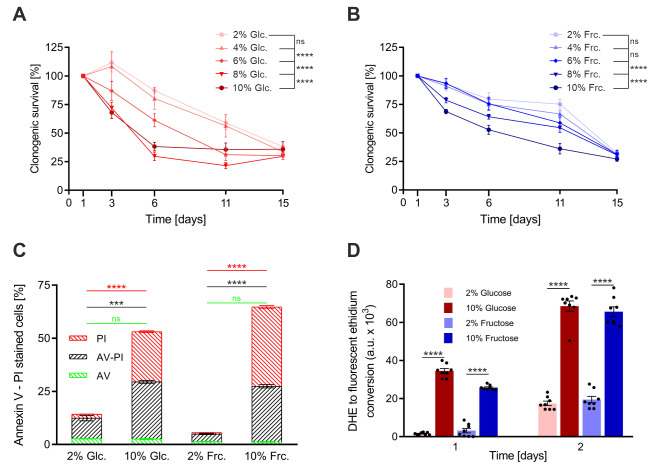
FIGURE 1: High glucose and fructose concentrations reduce CLS in yeast. Cultures of wild type (WT) cells were grown in synthetic complete media with either 2, 4, 6, 8 or 10% glucose **(A)** or fructose **(B)**. pH and osmotic effects were prevented by 25 mM sodium-acetate buffer and according amounts sorbitol supplementation (up to 8% sorbitol to gain same-level osmotic conditions in all different sugar concentrations used), respectively. Survival was determined by evaluating clonogenic capacity of 500 cells plated on YPD plates at indicated time points. Data represent the mean of 4 independent experiments. Graphs represent means of 3 independent experiments with error bars indicating ±S.E.M. Two-way ANOVA with a Bonferroni post hoc test was performed to determine p values: * p<0.05, ** p<0.01, *** p<0.001, **** p<0.0001 (A and B). **(C)** High glucose and fructose concentrations lead to primary necrotic and late apoptotic/secondary necrotic cell death within three days. The type of cell death was determined with Annexin V/ PI co-staining and subsequent flow cytometric analysis of WT cells on day 3 of chronological aging. **(D)** Glucose- and fructose-mediated cell death is accompanied by elevated ROS levels. ROS levels of WT cells were determined by conversion of DHE to fluorescent ethidium. Graphs represent means of 3 independent experiments with error bars indicating ±S.E.M. Multiple t test with correction for multiple comparisons using the Holm-Sidak method was performed to determine p values: * p<0.05, ** p<0.01, *** p<0.001, **** p<0.0001 (C and D).

First, we established the cytotoxic effects of glucose and fructose overload on yeast cells during chronological aging. As a control concentration, we used that of standardized minimal medium (2%). Yeast cells challenged with media containing increasing levels of glucose or fructose (4 to 10%) showed decreased cell survival over time, as determined by clonogenicity (**Fig. 1A, B**). Notably, glucose exerts toxic effects at lower concentrations than fructose. To limit unspecific effects, differences in acidification and osmolarity were buffered with a 25 mM sodium acetate buffer (in all conditions) and by addition of sorbitol (to the 2% control), respectively (Fig. S1A).

Metabolites released into the medium during sugar-stress, such as ethanol and acetate, might act as confounding factors. To test this, we exchanged the supernatants of 2% and 10% SMD cultured yeast after three days of chronological aging and followed survival. No differences were observed between the shifted samples and the non-shifted controls, indicating that no exogenous products are responsible for the observed lethal effects (Fig. S1B). Furthermore, we evaluated if other media components might limit high/complete sugar-uptake from the medium and thus be responsible for the shortened CLS. Hence, we measured sugar uptake from the medium with the highest glucose (10%) concentration. Glucose was taken up from the medium entirely within forty hours, which is in agreement with published data (Fig. S1C) 37,38.

To characterize the type of cell death induced by glucose- and fructose-stress during aging, we co-stained yeast cells with FITC-associated Annexin V (AV) and propidium iodide (PI) and quantified the different subpopulations using flow cytometry. AV binds to phosphatidylserine, which is externalized to the outer leaflet of the cell membrane upon apoptotic stimuli. PI is a DNA intercalator, which is only able to trespass the cell membrane if it is ruptured, *i.e.*, under necrotic conditions. AV- and PI-single stained cells thus represent the apoptotic and primary necrotic fractions respectively, while the AV/PI co-stained cells show the late apoptotic or secondary necrotic population, reflective of the fact that in yeast, apoptotic cells eventually become necrotic 39. Comparing 10% glucose- and fructose-stressed yeast to cells grown in standard medium at day three of chronological aging. For both sugars, we observed a large increase in the primary necrotic and late apoptotic/secondary necrotic subpopulations, which reached >50% (compared to 5-10% in the controls) (**Fig. 1C**). This was accompanied by markedly elevated levels of ROS, as measured by the conversion of dihydroethidium (DHE) to fluorescent ethidium (**Fig.1D**). Altogether, these results show that both glucose and fructose overload have detrimental effects during yeast chronological aging.

### Tor1 and Vac8 are involved in high-sugar load-mediated cytotoxicity

**Figure 2:**
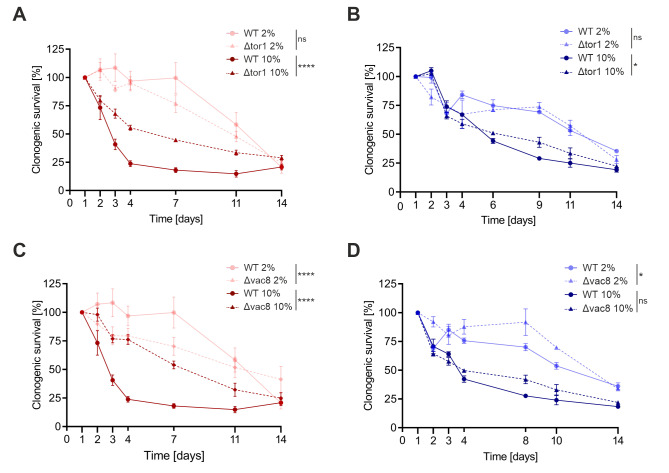
FIGURE 2: Deletion of either *TOR1 *or* VAC8* improves yeast CLS under high sugar conditions. Wild type (WT) and mutant strains Δ*tor1* **(A, B)** and Δ*vac8* **(C, D)** were cultured in 2% and 10% SMD **(A, C)** or 2% and 10% SMF **(B, D)** media supplemented with 25 mM sodium-acetate buffer as well as sorbitol. Survival was determined by evaluating clonogenic capacity of 500 cells plated on YPD plates at indicated time points. Graphs represent means of 3 independent experiments with error bars indicating ±S.E.M. Two-way ANOVA with a Bonferroni post hoc test was performed to determine p values: * p<0.05, ** p<0.01, *** p<0.001, **** p<0.0001.

In order to explore the mechanistic basis for the observed high-sugar cytotoxicity during chronological aging, we next evaluated a possible involvement of the most prominent yeast nutrient signalling pathway, which involves the master regulator kinase Tor1 (TOR-pathway). Compared to the wild type controls, *TOR1* deletion mutants showed a pronounced improvement in cell survival when challenged with a high glucose concentration (10%) (**Fig. 2A**). This effect was less prominent but still significant when cells were grown under high fructose (10%) conditions (**Fig. 2B**). Of note, glucose uptake from the medium was comparable in the mutant and the wild type strains, thus ruling out differences in glucose availability of the mutant strains (Fig. S2C).

We next tested other known downstream factors of the Tor-pathway, including components of both the autophagic and vesicle trafficking (transport and fusion) machineries. Autophagy is an intracellular degradation pathway that is activated in response to nutrient limitation and can be non-selective or selective. In yeast, one type of selective autophagy is the so-called cytoplasm to vacuole targeting (Cvt) pathway, which drives the vacuolar delivery of the hydrolase aminopeptidase or alpha mannosidase. We thus generated mutants carrying single gene deletions of the autophagic core machinery (Δ*atg5*, *7*, *8*), specific genes implicated in the Cvt pathway (Δ*ape1*, Δ*lap3*) as well as Δ*vac8*, which was originally thought to be specific for the Cvt pathway but has been shown to be involved in the general tethering of the Atg1 kinase complex and thus plays a role in piecemeal autophagy and various selective autophagies 40,41, and tested them under high glucose conditions during chronological aging. Compared to the wild-type strain, only the Δ*vac8* mutant showed improved survival (**Fig. 2C** and **D**) while all other deletion strains displayed more or less unaltered or worse survival under high glucose conditions (Fig. S2A and B). Importantly, the deletion of *VAC8* partially rescued high fructose toxicity, although less prominently. Of note, the Δ*vac8* mutant cells showed improved survival under fructose control conditions. In addition, the reduced survival of the autophagy core machinery deletion mutants under high glucose conditions (compared to wild type) argues for vital functions of autophagy under sugar stress. Taken together, *TOR1* and *VAC8*, were identified to take critically part in fructo- and glucotoxic cell death and were thus investigated further.

### Glucose and fructose overload result in elevated levels of neutral lipids and specific phytoceramide species.

**Figure 3:**
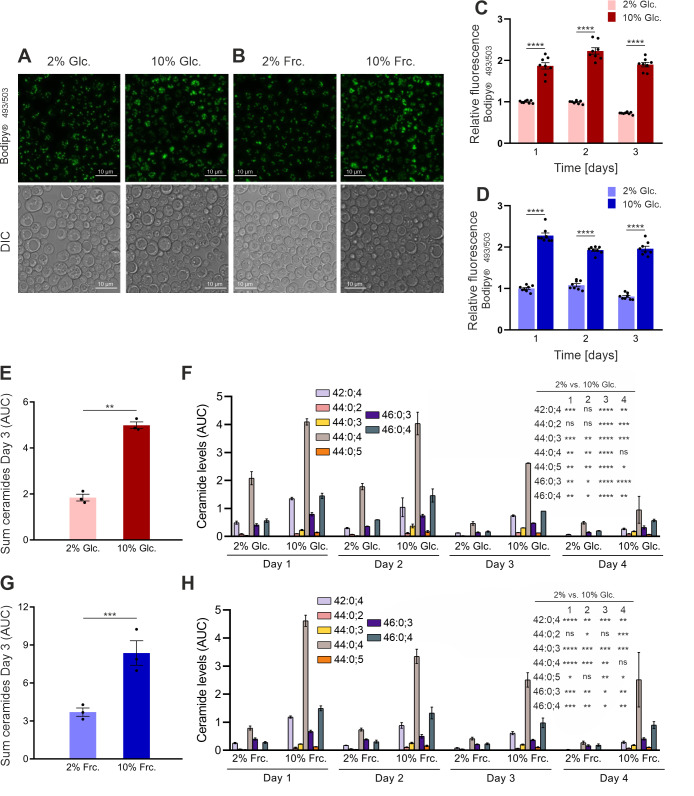
FIGURE 3: Analysis of glucose- and fructose-stressed wild type yeast cells shows specific elevation in the levels of neutral lipids, ceramides and an altered C_26_-phytoceramide profile. **(A and B)** Visualization of lipid droplets in wild type cultures upon Bodipy® 493/503 staining and subsequent confocal laser scanning microscopy of day 3 CLS- samples. 1^st^ row: Maximum projection of z-stacks. 2^nd^ row: DIC; Imaging was performed within the dynamic range. **(C and D)** Bodipy® 493/503 staining and subsequent FACS analysis of CLS cultures at given time points. **(E-H)** Ceramide content of 2% and 10% SMD/F-cultured BY4741 cells was analyzed via MS. High-sugar cultures showed elevated ceramide (SMD **(E)**), SMF **(F)** and specific phytoceramide species (SMD **(G)**, SMF **(H)**) levels. AUC were normalized to an internal standard. Graphs represent means of 3 independent experiments with error bars indicating ±S.E.M. Multiple t test was performed to determine p values: * p<0.05, ** p<0.01, *** p<0.001, **** p<0.0001.

To further validate our model, we next exerted if sugar overload leads to an elevation of the lipid content, an observation that has already been made in various organisms, including yeast and humans 8,9,42,43. In addition, various lipid species can modify vesicular membrane composition and thus affect fission and fusion events such as during vesicle trafficking 44,45,46,47,48, which given our results with Δ*vac8* seemed another hint to pursue this hypothesis. We therefore first evaluated the storage lipid content under our conditions by using Bodipy 493/503, a well-established fluorescent dye that mainly stains neutral lipid species accumulating in lipid droplets. Microscopic analysis of Bodipy 493/503-stained cells previously grown under high glucose or fructose concentrations (10%) both showed an elevated content of lipid droplets compared to the 2% standard conditions (**Fig. 3A** and **B**). In line, FACS analysis of these Bodipy 496/503-treated cultures confirmed significantly elevated neutral lipid content of cells grown under 10% glucose or fructose conditions (**Fig. 3C** and **D**). Prompted by these differences, we performed MS analysis of the whole cell lipid-content, which revealed a significant increase of DAG (Fig. S3A), TAG (Fig. S3B) and ceramides (**Fig. 3E** and **G**) upon sugar overload. Interestingly, phosphatidylcholine (PC) and phosphatidylethanolamine (PE) levels were reduced upon growth in high sugar media, indicating, that lipids are actively stored as neutral lipids or directed into ceramide synthesis upon high sugar (Fig. S3C and D). Of note, the ceramide levels (combining all ceramide species) were generally lower in glucose- than in fructose-grown cells, but the relative increase under high concentrations was similar in both cases (**Fig. 3E** and **G**). Analysis of ceramide species revealed C_26_-phytoceramides were specifically increased, with species C_26_-phytoceramide (44:0:4) having the most prominent increase under both high sugar conditions (**Fig. 3F** and **H**).

### The geroprotective effects of Δ*tor1* and Δ*vac8* upon sugar stress are linked to an overall reduction of ceramide levels and specific phytoceramide species

**Figure 4:**
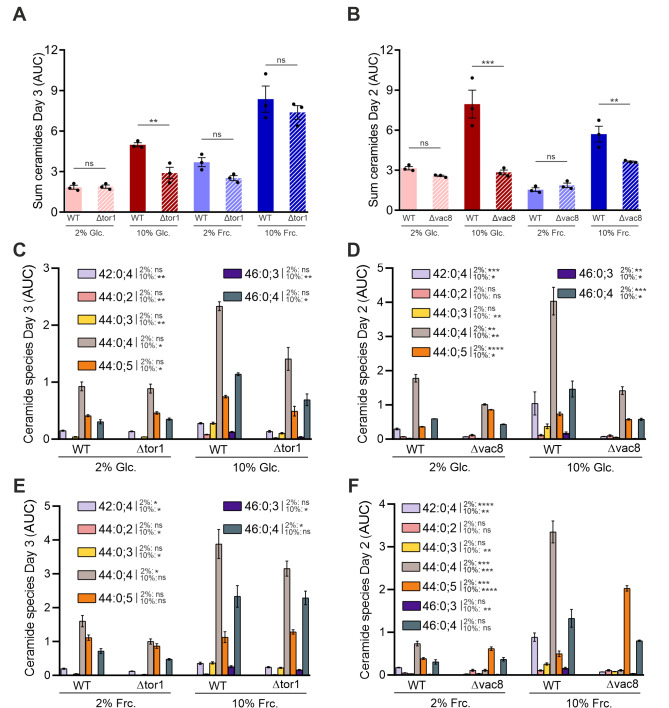
FIGURE 4: Δ*tor1 *and Δ*vac8 *show reduced ceramide levels overall and alterations of specific phytoceramide species under sugar overload. Sum of all ceramides in WT and Δ*tor1* **(A)**, and Δ*vac8* **(B)** cells cultured in SMD and SMF, respectively. Specific ceramide profiles of 1 x 10^9^ WT, Δ*tor1* **(C and E)**, and Δ*vac8* **(D and F)** cells cultured in 2% and 10% SMD/SMF. AUCs were normalized to internal standard IS C17:0. Graphs represent means of 3 independent experiments with error bars indicating ±S.E.M. Multiple t test was performed to determine p values: * p<0.05, ** p<0.01, *** p<0.001, **** p<0.0001.

Since ceramides were specifically altered in response to sugar-stress, we next analyzed ceramide levels in the two mutants we had identified to rescue gluco- and fructotoxicity. Intriguingly, Δ*tor*1 and Δ*vac8* strains both displayed a significant reduction of ceramide levels overall on both high glucose and high fructose media compared to the wild type control (**Fig. 4A** and **B**). The subsequent evaluation of specific ceramide species exhibited considerable changes compared to the wild type strain. When grown under high sugar conditions both Δ*tor*1 and Δ*vac8* showed a significant reduction of the most prominent C_26_-phytoceramide species 44:0:4, albeit the effect was more pronounced on glucose compared to fructose media (**Fig. 4C-F**). Also, the other tested ceramide species were mostly downregulated to different degrees compared to the wild type strain ceramide levels (**Fig. 4C-F**). Altogether, in particular phytoceramide species seem to be involved in the Tor1- and Vac8-mediated gluco- and fructotoxicity during chronological aging.

### Pharmacological inhibition of ceramide biosynthesis reduces cytotoxicity while its accumulation elevates cytotoxicity

To causally test the involvement of ceramides in cytotoxicity, we specifically reduced the overall content of sphingolipids in stressed and unstressed yeast cells by supplying myriocin, a specific inhibitor of serine palmitoyltransferase (*SPT1*), catalyzing the first and rate-limiting step in sphingosine biosynthesis 49,50.

**Figure 5:**
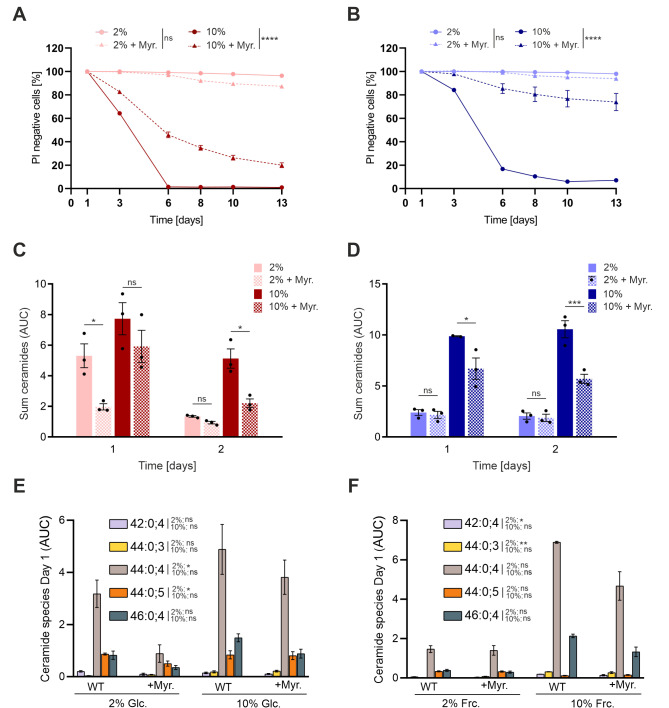
FIGURE 5: Myriocin treatment improves CLS and reduces ceramide levels upon sugar overload. CLS of cells grown in 2 and 10% SMD **(A)** and SMF **(B)** and supplemented with myriocin (Myr) where indicated. Graphs represent means of 3 independent experiments with error bars indicating ±S.E.M. Sum of ceramides in SMD **(C)** and SMF **(D)** media; levels were determined by MS analysis. Ceramide profiles of 1 x 10^9^ WT cells cultured in 2% and 10% SMD/ SMF **(E and F)**. Graphs represent means of 3 independent experiments with error bars indicating ±S.E.M. Two-way ANOVA with a Bonferroni post hoc test (A, B) and Multiple t test (C-F) was performed to determine p values: * p<0.05, ** p<0.01, *** p<0.001, **** p<0.0001.

**Figure 6:**
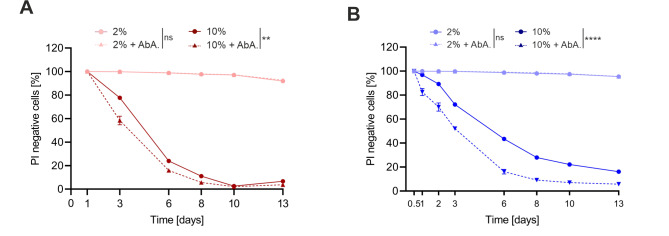
FIGURE 6: Administration of Aureobasidin A shortens the CLS of yeast. **(A, B)** CLS of cells grown in 2 and 10% SMD **(A)** and SMF **(B)** media supplemented with 25 mM sodium-acetate buffer as well as sorbitol and Aureobasidin A (AbA) where indicated. Survival was determined by PI-staining and subsequent FACS analysis of 30,000 cells at indicated time points. Graphs represent PI-negative cells of means of 3 independent experiments with error bars indicating ±S.E.M. Two-way ANOVA with Tukey's multiple comparisons test was performed to determine p values: * p<0.05, ** p<0.01, *** p<0.001, **** p<0.0001.

Myoricin treatment improved CLS during both glucose- and fructose-stress (**Fig. 5A** and **B**). In addition, overall ceramide levels were significantly reduced, while individual ceramide species, that were observed to increase with sugar toxicity including C_26_-phytoceramide (e.g. 44:0:4), where mildly reduced (**Fig. 5C** to **F**). Of note, myriocin has been well described to reduce (phyto-)ceramides specifically 50,51. In contrast, additional (phyto-)ceramide accumulation through downstream inhibition of complex ceramide synthesis using Aureobasidin A (AbA) 52,53, further exacerbated cell death under high-sugar conditions (**Fig. 6A** and **B**). Together these results argue for a major role of ceramides in the lipid-associated gluco- and fructotoxicity during chronological aging.

## DISCUSSION

Mechanistically, the influence of sugar overload on cell metabolism is multifaceted and remains largely understudied. The synthesis of (phyto-)ceramides, involves several steps including the condensation of serine and palmitoyl-CoA to produce 3-ketodihydrosphingosine, which is then further modified to form long-chain bases (LCBs) and eventually ceramides. Sugar metabolism is directly connected to the *de novo* ceramide biosynthesis through the availability of precursors such as acetyl-CoA. It is a central metabolite in sugar metabolism and is crucial for the synthesis of fatty acids which subsequently is used for the production of complex sphingolipids, including (phyto-)ceramides 54,55. A study in yeast demonstrated that reducing glucose concentrations in the culture medium led to a nearly sevenfold decrease in LCBs and their phosphorylated forms (LCBPs), which are precursors in ceramide biosynthesis. This suggests that glucose levels may influence ceramide synthesis by altering the availability of these precursors 56. Here, we identified molecular determinants of high mono-carbohydrate cytotoxicity by using a tightly controlled yeast model.

First, our data suggest fructo- and glucotoxicity to be dependent on the vesicle fusion machinery, specifically on Vac8. Vac8 is not only a critical component of the Cvt machinery, which comprises a biosynthetic route that makes use of the autophagic machinery, but is also required for vacuole nucleus junctions (NVJ) by direct interactions between Vac8 on the vacuolar and Nvj1 on the nuclear membrane as well as for vacuole-to-vacuole fusion events 57,58,59. Several studies indicate that NVJs facilitate lipid exchange and signalling between the vacuole and the ER, the primary site of sphingolipid biosynthesis, including ceramides. Disruption of Vac8 reduces NVJ formation and could therefore lead to altered lipid flux and ceramide transport from the ER to vacuole or Golgi, potentially affecting ceramide biosynthesis and accumulation 60,61,62. Furthermore, Vac8 and Nvj1 mediate a form of selective piecemeal autophagy, piecemeal microautophagy of the nucleus (PMN). The function of PMN is to degenerate and recycle non-essential portions of the nucleus and is independent of macroautophagy 58. Two membrane proteins with roles in lipid biosynthesis, Tsc13 and Osh1, are concentrated in NVJ-associated ER 63,64. Whether ceramides are also involved is not yet clear, but future work could provide a link between Vac8, PMN and elevated ceramide content in sugar-stressed cells. Vac8 is also involved in autophagosome formation. As autophagy affects lipid homeostasis by degrading organelles and membranes and recycling fatty acids and sphingolipids, deletion of Vac8 could reduce the turnover of complex sphingolipids, leading to altered ceramide levels 65,66. In addition, Vac8 also interacts with Ldo proteins to form the vacuole-lipid droplet contact site (vCLIP), which plays a critical role in lipophagy, so deletion of Vac8 potentially also affect lipid turnover and homeostasis in this way 67,68.

Our results further demonstrate a critical involvement of Tor1, the main component of the target of rapamycin (TOR) signaling pathway and TOR-complex 1 (TORC1): Tor1 deletion partly rescues the cytotoxic effects upon fructose and glucose stress. The TOR signaling pathway, which is conserved between yeast and mammalian cells 69, regulates anabolic processes like protein synthesis 70,71 and, importantly, inhibits catabolic processes like autophagy. In turn, dietary or pharmacological inhibition of TORC1 induces macroautophagy 71,72,73, which has been implicated in glucose-mediated ROS (superoxide) generation and effective cell cycle arrest 23,74. In collaboration with Tor1, the serine/threonine kinase Sch9 is also critical for the cellular response toward high-sugar growth conditions 23,74.

Interestingly, both Tor1 and Sch9 have also been implicated in sphingolipid metabolism, for instance in connection to Isc1 (inositol sphingolipid phospholipase C), the yeast orthologue of the mammalian sphingomyelinase 2. Isc1 is mitochondrially localized and hydrolyzes complex sphingolipids to produce phytoceramides. Upon deletion of *ISC1*, mitochondria display significantly lower phytoceramide levels 75, and cells show reduced CLS, higher ROS sensitivity, defective mitochondria and might also be impaired for autophagy. Double knockout of either *ISC1 *and *TOR1* or *ISC1* and *SCH9* restores mitochondrial function, lowers oxidative stress sensitivity and restores CLS impairment 76. Furthermore, the TORC1-Sch9 signaling pathway represses expression of *YDC1* and *YPC1*, the two genes encoding the ceramidases that generate phytohydrosphingosin from C 26- phytoceramide (44:4) 77.

Furthermore, TORC1 (as well as the TORC2) are involved in controlling sphingolipid synthesis by phosphorylating (either directly or indirectly) Orm1 and Orm2 - proteins that bind and control the serine palmitoyltransferase (SPT) as well as other enzymes of the sphingolipids/ceramide biosynthesis pathway. The inhibition of Tor signaling by rapamycin, causes hypophosphorylation of Orm proteins which leads to stronger inhibition of SPT, reducing flux through the sphingolipid biosynthetic pathway 78,79. Of further note, an indirect effect on TORC1 signaling may also occur, given the established role of the vacuole as a platform for TORC1 activation in yeast and the function of Vac8 as a major vacuolar membrane protein 80. Lipids are evolutionary conserved for energy storage and serve as pool for membrane generation in yeast 43. Across all species, it seems a conserved principle to store excess sugar in form of lipids. With our sugar-toxicity model we recapitulated the conversion of sugar-overload into elevated levels of DAG and TAG in the yeast *S. cerevisiae*.

However, it is noteworthy that we not only observed increased levels of neutral lipids but also (specific) ceramides following fructose and glucose overload, while PE and PC levels remained relatively unchanged. Ceramides, a subclass of sphingolipids composed of a sphingosine backbone and a fatty acid chain 81,82 have already been shown to act as signaling molecules for cell growth/differentiation, cell cycle arrest, stress response, endocytosis, lifespan-regulation and apoptosis 83,84,85,86,87. Published data indicate that caloric restriction and exercise lead to depletion of ceramides (and other sphingolipids), which is consistent with the growing notion that ceramides have major implications in the health status of a person 88,89. In yeast, lowering the concentration of sphingolipids (e.g., via myriocin treatment) leads to a significant improvement in CLS in a similar way than reducing the activity of TORC1 or Sch9. In fact, there even seems to be a synergistic effect upon simultaneous treatment of wild type cells with rapamycin (TORC1 inhibitor) and myriocin as well as of Δ*sch9* cells with myriocin 86.

In our experimental model, the general increase in ceramide levels and, particularly, that of specific phytoceramide species might thereby be causally linked to fructo- and glucotoxicity during chronological aging. In fact, cellular protection via either genetic (Δ*tor1* and Δ*vac8*) or pharmacological means (myriocin treatment) was accompanied by reduced ceramide levels and a decrease in those phytoceramide species that were specifically enhanced upon sugar toxicity, including C_26_-phytoceramide 44:4. Intriguingly, strains blocked in the TORC1-Sch9 signaling pathway showed upregulated activity of *YDC1* and *YPC1 *promotors 77 and thus would lead to depletion of both the C_26_-phytoceramide pool (see previous paragraph), which aligns well with our results.

(Phyto-)Ceramides have been directly implicated in yeast regulated cell death, more specifically in mitochondria-controlled demise 90,91. The importance of mitochondria is conserved in higher eukaryotes, where, for instance, ceramides were shown to trigger mitochondrial apoptosis by binding the voltage-dependent anion channel VDAC2 92. Further, ceramides have been recognized as tumor suppressor lipids and key mediators of anti-proliferative and tumor-suppressive cellular programs, which - besides apoptosis - include mitophagy, cell cycle arrest, and senescence 93,94. Finally, ceramides have long been connected to aging regulation 95,96. In yeast, deletion of Sit4, the catalytic subunit of a ceramide-activated PP2A-like phosphatase (that regulates cell cycle, mitochondrial function, oxidative stress resistance and chronological lifespan in yeast), increases CLS 97. In turn, Sit4 activation has been associated with a shortened lifespan 98. In addition, low doses of myriocin additionally prolong yeast CLS due to global changes in gene expression that have an impact on several pathways: activation of the Snf1/AMPK pathway, downregulation of the PKA, TORC1, and the sphingolipid-controlled Pkh1/2-Sch9 pathways. Thus, this treatment mimics the effects of rapamycin administration or caloric restriction 99.

Phytoceramides are the predominant ceramide species of yeasts (as well as of plants) and have specific biophysical characteristics that partly differ from ceramide and di-hydroceramide species 100,101. Therefore, it remains to be tested whether the observed effects recapitulate some of the pathophysiological roles of (other) ceramide levels and species that have been associated with human conditions such as Alzheimer ’s disease, cardiometabolic diseases, cancer, or hepatosteatosis 102,103,104,105,106,107. Furthermore, there are emerging evidences of high sugar diets leading to critical ceramide levels in general or even of specific species, corroborating our results and showing its potential relevance for human pathologies 103,108. Beyond these "modeling aspects", even effects that are specific to phytoceramides might play a critical role in human organ systems, where phytoceramides are present in high abundance such as the intestinal tract, the kidney and the skin. Here, the insight of a critical involvement of phytoceramides in (various) pathological conditions has only recently emerged 109,110.

Further it could be shown that dysregulation of diverse sphingolipid species influences mitochondrial functions, oxidative stress resistance and CLS 111,112. In mammals, accumulating evidence points to the engagement of sphingolipids in several aging-related processes 113,114, including brain aging and the onset/prevalence of neurodegenerative disorders such as Alzheimer's, Parkinson's, and Huntington's disease as well as amyotrophic lateral sclerosis 115,116. In primary cells such as mouse embryonic fibroblasts (MEFs), it could be demonstrated that dysregulation of ceramide transfer from the ER to the Golgi apparatus compromise cellular fitness, mainly through an impact on mitochondria, leading to premature senescence 117.

We have previously shown that DAG levels influence yeast regulated cell death (RCD) and CLS 118. The herein presented results add to these observations and highlight the importance of (phyto-)ceramides for gluco- and fructotoxicity. Of note, changes in DAG and TAG contents observed in Δ*tor*1, were not down-regulated consistently. While TAG contents were up- or down-regulated DAG was down-regulated overall (Fig. S4A and B). In addition, a deletion of *PAH1*, a phosphatidate phosphatase that catalyses the dephosphorylation of phosphatidate to yield DAG and which has already been shown to lead to diminished DAG levels 119,120, still exerts significant sugar-induced shortened CLS (Fig. S4C and D).

Of note, deletion of *PAH1 also* leads to a reduction of phosphoinositol-containing sphingolipid content overall and significant alterations of specific phosphoinositol-containing sphingolipid species (e.g. downstream products of ceramides such as IPC, MIPC and M(IP)_2_C) which, potentially further links its effects on CLS under high sugar load to ceramides 121.

While we do not rule out that the sugar-mediated increase in DAG levels may contribute to gluco- and fructotoxicity, our results suggest that at least our observed effects are ceramide-specific. First, myriocin treatment, which specifically reduces sphingolipids and thus ceramides, improved CLS; secondly, ceramide accumulation upon AbA treatment promoted the negative effects of sugar on CLS; and third, the mutant strain Δ*pah1*, which exerts limited DAG synthesis, still displayed significantly elevated cell death under high-sugar conditions.

Altogether, our results establish a yeast model that allows for the exploration of sugar-induced cytotoxicity during post-mitotic aging. We have thereby identified molecular pathways and single transducers, most importantly ceramides that act as drivers of aging under sugar overload. These findings set the stage to define the executionary role of specific ceramide species in contrast to overall ceramide levels in order to devise possible new intervention strategies to tackle (age-related) conditions that are induced or amplified by excess sugar consumption.

## MATERIAL AND METHODS

### Yeast strains and media

All experiments were carried out in strains using the BY4741 wild type (MATa *his3*Δ*1 leu2*Δ*0 met15*Δ*0 ura3*Δ*0*) strain from EUROSCARF as basis (**Table 1**). In brief: Single gene deletions (*VAC8, PAH1*) were carried out by classical homologous recombination using the pUG vector systems 122,123 and controlled by PCR. Transformation was done using the lithium acetate method 124, giving rise to strains BY4741 Δ*vac8* and BY4741 Δ*pah1, *respectively*.* BY4741 Δ*tor1 *was constructed similarly 125. Notably, at least two different clones were tested for any experiment with these newly transformed strains to rule out clonogenic variation of the observed effect.

**Table 1:** Strains used for this study.

**Strain**	**Genotyp**	**Reference**
BY4741	MATa *his3*Δ*1 leu2*Δ*0 met15*Δ*0 ura3*Δ*0*	Euroscarf
BY4741 Δ*sch9*	BY4741 *SCH9*::kanMX	Euroscarf
BY4741 Δ*tor1*	BY4741 *TOR1*::kanMX	125
BY4741 Δ*vac8*	BY4741 *VAC8*::kanMX	this work
BY4741 Δ*pah1*	BY4741 *PAH1*::kanMX	this work
BY4741 Δ*atg5*	BY4741 *ATG5*::*URA3*	125
BY4741 Δ*atg7*	BY4741 *ATG7*::*HIS3*	this work
BY4741 Δ*atg8*	BY4741 *ATG8*::*HIS3*	this work
BY4741 Δ*ape1*	BY4741 *APE1*:kanMX	Euroscarf
BY4741 Δ*lap3*	BY4741 *LAP1*:kanMX	Euroscarf

Strains were grown on synthethic medium containing 0.17% yeast nitrogen base (BD Diagnostics; without ammonium sulfate and amino acids), 0.5% (NH_4_)_2_SO_4_, 30 mg/L of all amino acids (aa) (except 80 mg/L histidine and 200 mg/L leucine), 30 mg/L adenine, and 320 mg/L uracil supplemented either with glucose (SMD) or fructose (SMF) to the indicated final concentrations (2% or 10%). Unless otherwise indicated, growth media were stabilized with sodium-acetate buffer at a final concentration of 25 mM. To assure that the osmotic conditions are comparable in media with different sugar concentrations, the 2% SMD/F media were supplemented with according amounts of sorbitol. All amino acids were purchased from Serva (research grade, >=98.5%, Germany), D-Glucose was purchased from Neolab (Germany), D-Fructose was purchased from Sigma-Aldrich (USA) and Sorbitol was purchased form Fluka BioChemika (Germany). Survival plating was done on YPD agar plates (2% peptone, 1% yeast extract, 2% glucose, and 2% agar) and incubated for 2 days at 28°C.

### Chronological aging, clonogenicity assay and PI staining

The yeast strains were grown in overnight cultures (ONCs) in either 2% SMD or 2% SMF medium stabilized with 25 mM sodium-acetate buffer. The main cultures were inoculated to 10 mL SMD/F (in case strains carrying a plasmid the media lacked the corresponding amino acid) media, to an OD_600_ of 0.1 and incubated at 28°C with constant shaking. Sugar-stressed cells were cultured in 10% glucose containing SMD or 10% fructose containing SMF medium and survival was compared to non-stressed cells grown on SMD or SMF medium containing 2% of the corresponding carbon source. To assure that the osmotic conditions are comparable in both media, the 2% SMD/F media were supplemented with sorbitol. At indicated time points, aliquots were taken and survival was determined by clonogenic assays. Cell counts were determined with a CASY cell counter (Schärfe System, Germany), and 500 cells were plated on 2% YPD agar plates using appropriate dilutions. The plates were then incubated at 28°C for 2 days. Alternatively, cell viability was tested via propidium iodide (Pi) staining with subsequent flow cytometry analysis of 30,000 cells for each sample and data evaluation using the BD FACSAria instrument and BD FACSDiva software.

### Dihydroethidium to ethidium bromide conversion and Annexin V/ propidium iodide co-staining

ROS accumulation (super-oxide-anion) in cells was detected by the oxidation of non-fluorescent dihydroethidium (DHE; working concentration: 2.5 µg/mL in PBS; 5x10^6^ cells; incubation time 5 to 10 min at RT) to fluorescent ethidium bromide Annexin V/propidium iodide (Roche, Germany) staining was used to determine regulated cell death markers and necrosis (2x10^7^ cells; incubation time 5 to 10 min at RT) Experiments were quantified by using a fluorescent plate reader (Tecan, GeniusPRO) or by flow cytometry (BD FACSAria and BD FACS Diva software, 30,000 cells/sample) as previously described 125,126,127.

### Bodipy® 493/503 staining of neutral lipids/lipid droplets and FACS analysis/confocal fluorescence microscopy

Lipid droplets were visualized using Bodipy® 493/503, which stains neutral lipids, on a confocal fluorescence microscopy and quantified via flow cytometry analysis (FACS). Briefly: 5 x 10^6^ cells were harvested and resuspended in 250 µL 1xPBS/Bodipy® solution according to the manufacturers protocol (Life Technologies, USA). After 5 minutes incubation in the dark, cells were washed and analyzed. For fluorescence microscopy a Zeiss Axioskop fluorescence microscope, with a suitable band pass filter for GFP or RFP was used. The confocal fluorescence microscopy was performed on a Leica SP5 confocal microscope with spectral detection and a Carl Zeiss LSM510 with photomultiplier tubes. The neutral lipid marker BODIPY® 493/503 was excited at 488 nm and emission detected between 500 and 530 nm (spectral detector). The confocal fluorescence microscopy was performed in the dynamic range. For each flow cytometry analysis, 30,000 cells were analyzed and data was evaluated using the BD FACSAria instrument and BD FACSDiva software.

### Myriocin and Aureobasidin A treatment

During chronological aging, cultures were treated with 150 µg/mL (Sigma, USA; for CLS experiments to prevent slower cell growth and cell-clustering) or 450 µg/mL Myriocin (for lipid analysis) and 12.5 ng/mL Aureobasidin A (Sigma, USA), respectively, each added at inoculation of the main cultures.

### Lipid extraction and analysis

All protocols in this section followed the methods described previously 128.

#### Ceramide extraction

1 x 10^9^ cells were harvested, 25 µL of the internal standard mix were added to every sample. Next the yeast cells were homogenized in a 15 ml shot tube for 30 min at 4°C on the evapor mixer after addition of 150 µL glass beads and 2 mL 95 % ethanol: water: diethyl ether: pyridine: ammonium hydroxide in a ration 15:15:5:1:0.018 (v/v/v/v/v). After an incubation of 30 min at 60°C the homogenate was centrifuged at 3,500 rpm for 5 min. The supernatant was carefully transferred into a fresh Pyrex tube. Pellets were resuspended through addition of 2ml 95% ethanol: water: diethyl ether: pyridine: ammonium hydroxide in a ration 15:15:5:1:0.018 and incubation at 60°C for 20 min. Again, the homogenate was centrifuged at 3,500 rpm for 5 min and the supernatants were pooled. The aqueous phase was removed under nitrogen gas-stream. This protocol follows the method of Angus and Lester 129.

#### Mild Alkaline Hydrolysis for Neutral Lipids and Phospholipids 

Hydrolysis was performed in 15 mL Shot tubes by addition of 400 µL CHCl_3_:MeOH:water (16:16:5 (v/v/v)) and 400 µL 0.2M NaOH in Methanol (from a 2M NaOH Stock solved in water). After incubation at 30°C for 120 min 400 µL 0.5 M EDTA and 150 µL 1 N acetate solutions were added. 1 mL CHCl_3 _was added and lipids were extracted through vortexing. The aqueous phase was removed after centrifugation at 4,000 rpm for 2 min. 700 µL water was added. After vortexing and centrifugation (4,000 rpm, 2 min) the aqueous phase was removed and the organic phase evaporated under nitrogen gas-stream. Lipids were resuspended in 1 mL CHCl_3_:MeOH (2:1) and transferred to 2 mL vials. The organic phase was evaporated again under nitrogen gas-stream. The samples were stored at -20°C. This protocol follows the method of Guan and Wenk 130. Internal standards were as follows: DAG was normalized to DAG 28:0 and DAG 36:0. TAG was normalized to three different internal standards (TAG 45:0, TAG 51:0 and TAG 57:0). PC was normalized to PC 24:0 PC 34:0 and PC 38:0 as well as PE was normalized to PE 24:0 and PE 34:0.

#### UPLC-MS

Chromatographic separation was performed with an AQUITY-UPLC system (Waters, Manchester, UK) equipped with a BEH-C18-column (2.1 × 150 mm, 1.7 µm) and a HSS T3-column (100 x 2.1 mm, 1.8 µm) (Waters). The column compartment is kept at 50°C. A binary gradient was applied. Solvent A consisted of water/methanol (1:1, v/v), solvent B consisted of 2-propanol. Both solvents contained phosphoric acid (8 µM), ammonium acetate (10 mM) and formic acid (0.1 vol%). For the BEH-C18-column the linear gradient started at 45% solvent B and increased to 90% solvent B within 30 min. In the following 2 min solvent B percentage was increased to 100% and was kept at this level for 10 min. Starting conditions were achieved in 1 min and the column was re-equilibrated for 7 min, resulting in a total HPLC run time of 50 min. For the HSS T3-column the total separation time was 15 min. The linear gradient started at 45% solvent B and increased to 90% within 11 mins. It was then increased to 100% within the next 2.5 mins and then reduced to 45% for the next 2.5 mins. For analysis a SYNAPT™G1 qTOF HD mass spectrometer (Waters) equipped with an ESI source was used. Therefor following source parameters were set-up: capillary temperature 100°C, desolvatization temperature: 400°C, N_2_ as nebulizer gas. The capillary voltage was 2.1 kV in the negative ionization mode. For MSE two alternating scan modes were defined in the MS setup 131. The first scan mode resembles the full scan (mass range: m/z 50-1800; scan time: 1 s; data collection: centroid). The second scan mode (mass range: m/z 50-1800; scan time: 1 s; data collection: centroid) applied a collision energy ramp (25-45 V) to fragment all generated ions. Both scan modes showed a resolution of around 9,000 (FWHH). The lock spray was achieved by an external pump (L-6200, Hitachi) at a flow rate of 0.2 mL/min split in a 1:13 ratio. Leucine-enkephaline ([M + H]^+^: m/z 556.2771 and [M-H]^-^: m/z 554.2615) was used as reference substance in the lock-spray. The lock mass was measured every 15 s independent of the other scan modes, which allowed a continuous mass correction. The mass error was always below 5 ppm. As an internal standard a mix of 0.05 mg/mL C17:0 ceramide and 0.05 mg/mL C8:0 glycosyl-(β)-ceramide (Avanti Polar Lipids, USA) in chloroform/methanol 2:1 (v/v) was used. The MassLynx 4.1 software (Waters) was used for data acquisition. For further lipid analysis the "Lipid Data Analyser" software was used 132. The batch quantitation was done with the following setup: retention time tolerance before/after: 0.5 min; relative base peak cut off: 0.1‰; retention time shift: 0.5 min; isotopic quantitation of 2 isotopes where 1 isotopic peak has to match.

### Statistical analysis

To rebut the null-hypothesis significance and p-values were calculated using t-test and correction for multiple comparisons using the Holm-Bonferroni method with α = 0.05 for one variable (type of treatment) or a two-way ANOVA corrected by a Bonferroni post-hoc test for two variables (type of treatment and time). Significance is indicated with asterisks: *** p<0.001, ** p<0.01, * p<0.05. Statistics and figures were conducted with GraphPad Prism®6 software.

### Data availability statement

The data that support the findings of this study are openly available in Zenodo at http://doi.org/10.5281/zenodo.15745562

## CONFLICT OF INTEREST

The authors declare no conflict of interest. FM has equity interest in Samsara Therapeutics and TLL, The Longevity Labs GmbH.

## SUPPLEMENTAL MATERIAL 

Click here for supplemental data file.

All supplemental data for this article are available online at https://www.cell-stress.com/researcharticles/2025a-schmiedhofer-cell-stress/
