# Supplementary material for: Sugar accelerates chronological aging in yeast via ceramides
Source: Cell Stress. 2025 Jul 22;9:158–73. doi: 10.15698/cst2025.07.308 (PMC12318584; doi:10.15698/cst2025.07.308)
Supplement: Supplementary file 1 [file ces-09-158-s01.pdf]

Supplemental Material

Sugar accelerates chronological aging in yeast via ceramides

Vera Schmiedhofer[1,a], Julian Sommersguter-Wagner[1,a], Oskar Knittelfelder[1,2], Helmut Jungwirth[3], Gerald N. Rechberger[1,4], Didac Carmona-Gutierrez[1,4], Patrick Rockenfeller[5], Christoph Ruckenstuhl[1,4,*] and Frank Madeo[1,4,6,*]

[1] Institute of Molecular Biosciences, University of Graz, NAWI Graz, Graz, Austria. [2] Max Planck Institute of Molecular Cell Biology and Genetics, Dresden, Germany. [3] Center for society, knowledge and communication (7th faculty), University of Graz, Graz, Austria. [4] Field of Excellence BioHealth-University of Graz, Graz, Austria. [5] Chair of Biochemistry and Molecular Medicine, Center for Biomedical Education and Research (ZBAF), University of Witten/Herdecke (UW/H), Witten, Germany. [6] BioTechMed Graz, Graz, Austria.

* Corresponding Authors:

Christoph Ruckenstuhl, E-mail: ru.ruckenstuhl@uni-graz.at

Frank Madeo, E-mail: frank.madeo@uni-graz.at

[a] These authors contributed equally.

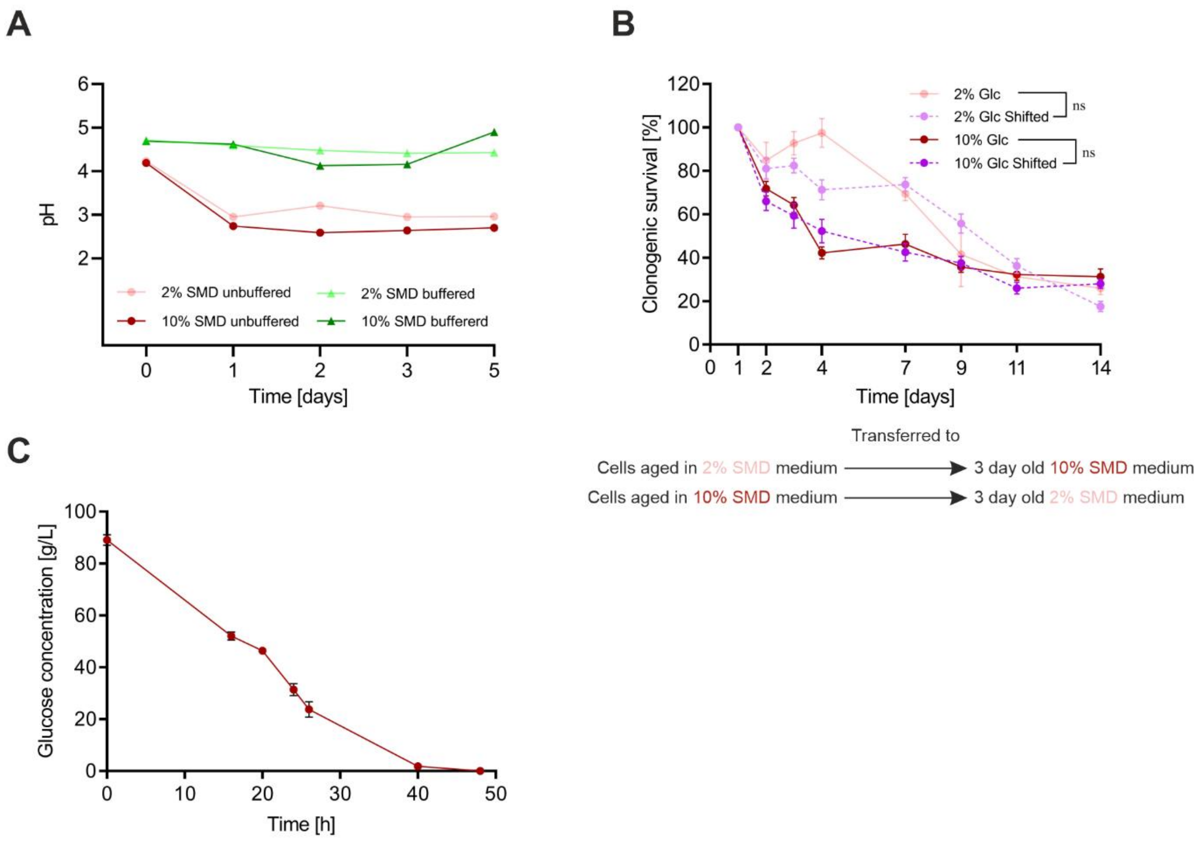

Supplemental FIGURE S1 ● **(A)** pH stabilization and osmolarity compensation. Overnight cultures of wild type cells (WT) were grown in SMD medium with 2% glucose. The main cultures were grown in buffered (i.e., addition of 25 mM sodium acetate buffer) and non- buffered SMD media over a time course of five days. Osmotic effects were prevented by according amounts of sorbitol supplementation (up to 8% sorbitol to gain same-level osmotic conditions in all different sugar concentrations used). In both media, the buffered samples stayed at a constant value of about 4.5, while in unbuffered conditions the pH dropped to 2.5. **(B)** A media switch between control and high glucose concentration does not influence survival during aging. Overnight cultures of WT cells grown in minimal complete medium with 2 % glucose, while the main culture was inoculated in minimal media with 10% and 2% glucose. After three days of chronological aging, cultures aged in 2% and 10% SMD were pelleted and the supernatants were exchanged, pellets resuspended and chronological aging resumed. Unshifted samples of both variants served as controls. Data represent the mean ± S.E.M. of surviving cells of 4 independent experiments. Two-way ANOVA with a Bonferroni post hoc test was performed to determine p values: * $p<0.05$, ** $p<0.01$, *** $p<0.001$, **** $p<0.0001$. **(C)** Glucose in 10% SMD is taken up by wt cells within fourty hours after inocculation. Overnight cultures of WT cells were grown in minimal complete media with 2% glucose, while the main culture was inoculated in minimal media with 10% glucose. The sugar concentration of the liquid medium was determined during a time course of forty-five hours. Data represent the mean of 3 independent experiments. Error bars indicate S.E.M.

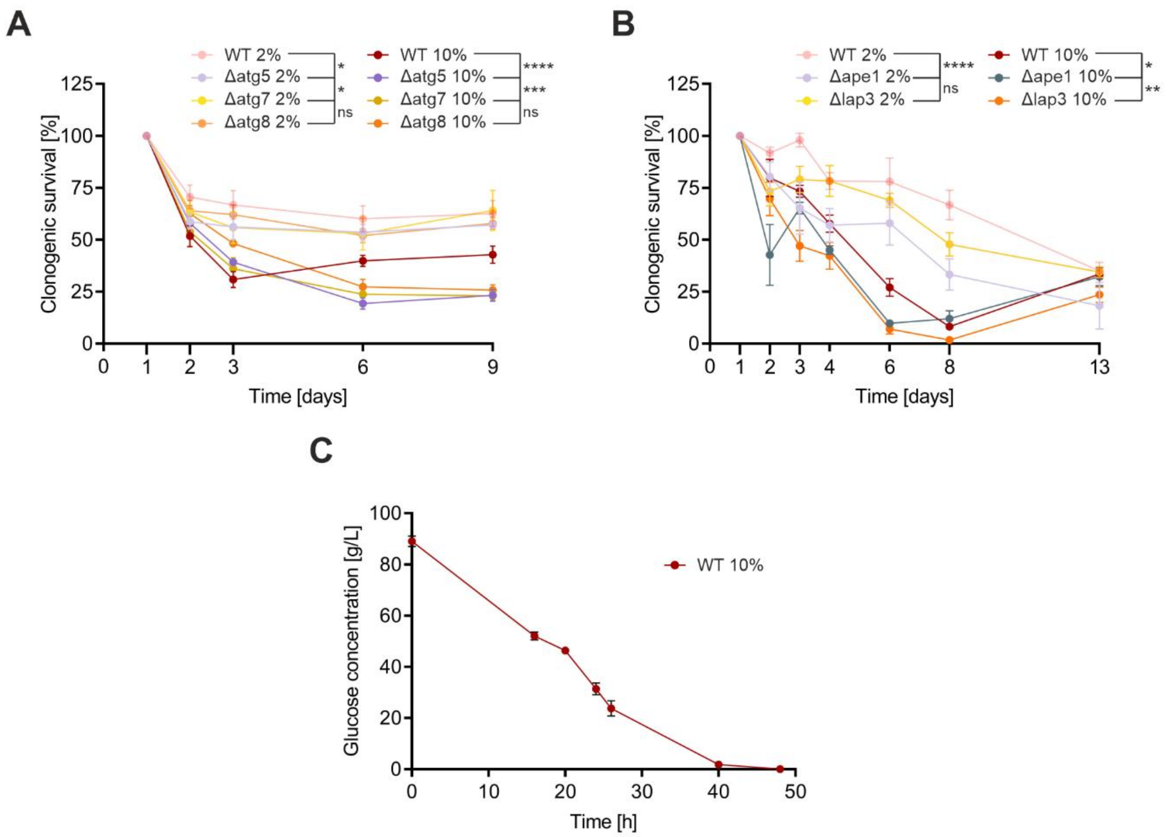

Supplemental FIGURE S2 ● **Evaluation of single-gene deletions of autophagy-related genes (A) and of essential genes of the cytoplasm-to-vacuole-targeting (CVT) pathway (B).** Wild type (WT) and mutant strains were cultured in 2% and 10% SMD medium, respectively. 25 mM sodium acetate buffer 25mM sodium-acetate buffer was added to avoid pH effects. Osmotic effects were prevented by sorbitol supplementation. The herein showed deletion mutants of autophagy related genes **(A)** and essential genes of the Cvt pathway **(B)** do not rescue glucose-mediated cell death. **(C)** Overnight cultures of BY4741 Δ*tor1* mutants were grown in SMD media with 2% glucose. The main culture was inoculated in synthetic media with 10% glucose. The sugar concentration of the liquid medium was determined during a time course of 45 hours. Graphs represent means of 3 independent experiments with error bars indicating ±S.E.M. Two-way ANOVA with a Bonferroni post hoc test was performed to determine p values: * $p<0.05$, ** $p<0.01$, *** $p<0.001$, **** $p<0.0001$.

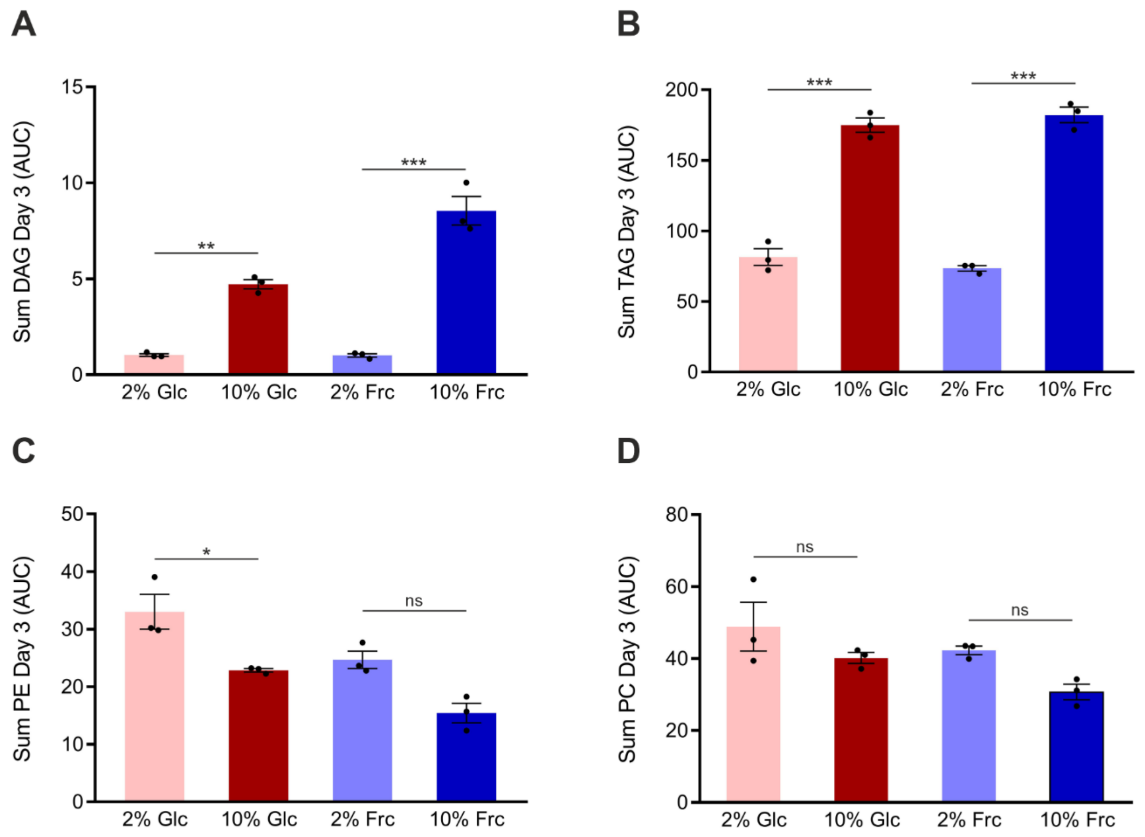

Supplemental FIGURE S3 ● **MS analysis of all measured lipid levels of yeast grown under high sugar levels shows alterations in DAG and TAG but not in PE and PC.** The lipid content of 2% and 10% SMD/F-cultured wild type cells was analyzed by MS-analysis of 1 x 10^9 cells. AUCs were normalized to an internal standard. High-sugar cultures showed increased levels of DAG **(A)** and TAG **(B)**, and showed overall a non-significant trend towards reduction in PE (phosphatidylethanolamine; **(C)**) and PC (phosphatidylcholine; **(D)**) content. Graphs represent means of 3 independent experiments with error bars indicating ±S.E.M. Multiple t test was performed to determine p values: * $p<0.05$, ** $p<0.01$, *** $p<0.001$, **** $p<0.0001$.

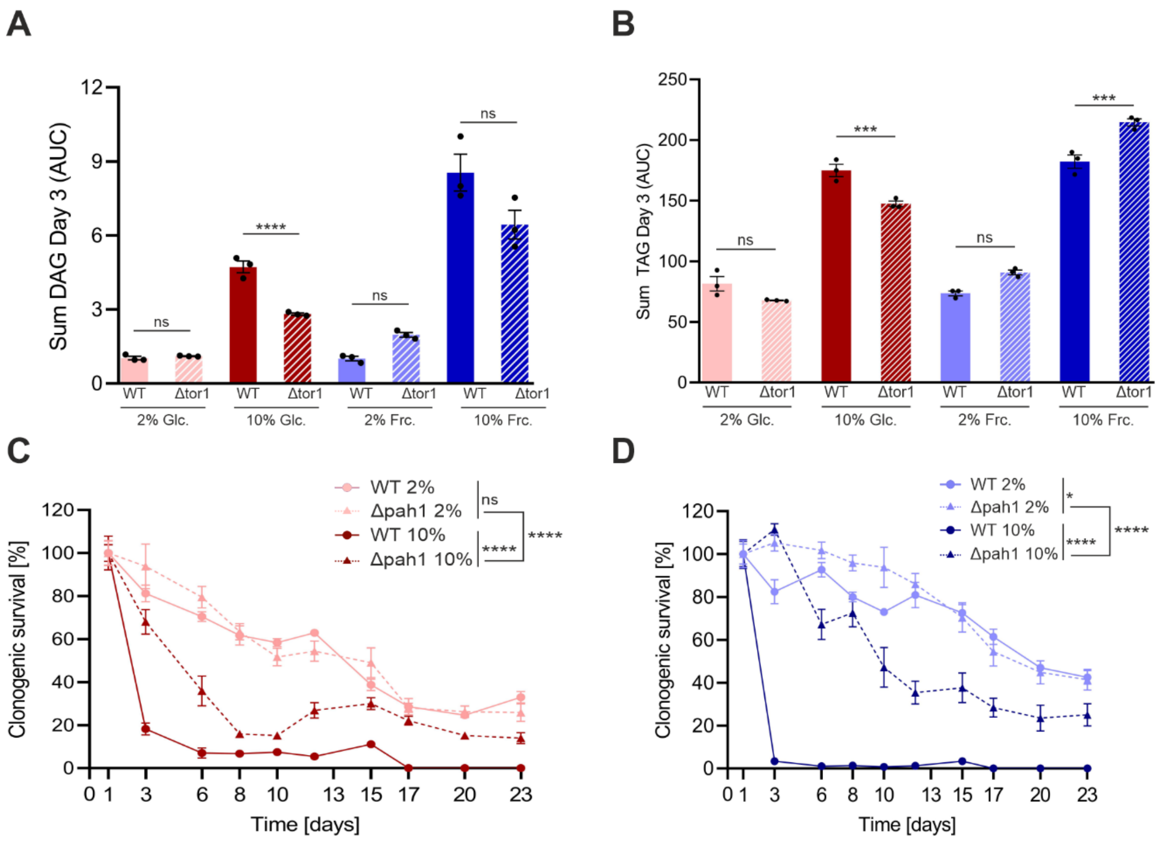

Supplemental FIGURE S4 ● DAG and TAG lipid levels of Δ*tor1* mutant strains as well as CLS of *pah1* mutants cultured in 10% SMD and SMF. (A) DAG and (B) TAG profiles of 1 x 10^9 wild type (WT), Δ*vac8* and Δ*tor1* deletion strains cultured in 2% and 10% SMD/ SMF. (C, D) CLS of cells grown in 2% and 10% SMD (A), or SMF (B). WT and mutant strains Δ*pah1* were cultured in 2% and 10% SMD (C) or 2% and 10% SMF (D) media supplemented with 25 mM sodium-acetate buffer as well as sorbitol. Survival was determined by PI-staining and subsequent FACS analysis of 30,000 cells at indicated time points. Graphs represent PI-negative cells of means of 3 independent experiments with error bars indicating S.E.M. Two-way ANOVA with a Bonferroni post hoc test (A, B) or Tukey's multiple comparisons test (C, D) was performed to determine p values: * $p<0.05$, ** $p<0.01$, *** $p<0.001$, **** $p<0.0001$.
